# Enhancement of the water-resistance properties of an edible film prepared from mung bean starch via the incorporation of sunflower seed oil

**DOI:** 10.1038/s41598-020-70651-5

**Published:** 2020-08-12

**Authors:** Jung-Soo Lee, Eun-sil Lee, Jaejoon Han

**Affiliations:** 1 Department of Biotechnology, College of Life Sciences and Biotechnology, Korea University, Seoul, 02841 Republic of Korea; 2 Department of Food Biosciences and Technology, College of Life Sciences and Biotechnology, Korea University, Seoul, 02841 Republic of Korea

**Keywords:** Materials chemistry, Polymer chemistry

## Abstract

Mung bean starch (MBS)-based edible films with incorporation of guar gum (GG) and sunflower seed oil (SSO) were developed in this study. MBS, GG, and SSO were used as the main filmogenic biopolymer, thickener, and hydrophobicity-imparting substance, respectively. To investigate the effect of SSO content on the physicochemical, mechanical, and optical properties of the films, they were supplemented with various concentrations (0, 0.5, 1, and 2%, w/w) of SSO. Increasing SSO content tended to decrease tensile strength, elongation at break, crystallinity, water solubility, and the water vapor permeability; in contrast, it increased the oxygen transmission rate and water contact angle. Consequently, the incorporation of SSO into the matrix of MBS-based films decreased their mechanical strength but effectively enhanced their water-resistance properties. Therefore, the MBS-based film developed here can be properly used as an edible film in settings that require high water-resistance properties but do not call for robust mechanical strength.

## Introduction

Edible films and coatings improve the quality of foods by protecting them from physicochemical and biological deterioration. Examples of such deterioration events include weight loss or gain, the disappearance of the original texture, discoloration, flavor change, and growth of microorganisms. The main ingredients of edible films and coatings are edible filmogenic biopolymers, such as proteins, polysaccharides, lipids, or combinations of these molecules. Plasticizers, cross-linkers, and other food-grade additives are combined with the filmogenic biopolymers to modify the properties or functionalities of the edible films and coatings^1^. Edible films and coatings have been studied extensively using various materials to improve food quality. For example, the application of a carboxymethyl-cellulose-based coating onto beef patties suppresses lipid oxidation and microbial growth^2^. Waxy corn starch- and gellan gum-based coating on rice cakes delays their retrogradation (hardening) and helps maintain their chewy texture^3^. Soy protein coating on walnut kernels retards lipid oxidation^4^. Carnauba wax-based coating on eggplants helps maintain firmness, color, and antioxidant activity^5^. Similarly, many different ingredients are used for manufacturing edible films and coatings.

Among these diverse sources of materials, starch is the most significant polysaccharide polymer because it has a superb ability to form a compact, homogenous, and continuous matrix. Therefore, starch-based films have high oxygen-barrier ability and good mechanical strength. In addition, starch is renewable, biodegradable, non-toxic, tasteless, odorless, colorless, abundant, and low in cost^6,7^. Starchy foods include potatoes, corn, peas, wheat, and bananas.

Mung beans (*Vigna radiata* L.) are starch-rich foods. Mung bean starch (MBS) has a high amylose content (30–45%)^8^, which is higher than that of cereals and tubers^9^. Amylose content in the starch is a significant factor in determining the mechanical and resistance characteristics of the starch-based films. The linear structure of the amylose forms a dense network in a polymeric structure. In contrast, branched amylopectin produces a less dense network compared with amylose. Therefore, the strength and resistance properties of amylose-based films are, in general, better than those of amylopectin-based films^10^. For this reason, MBS with a high amylose content was chosen as a filmogenic biopolymer for the development of starch-based edible films in this study.

Starch-based films have one major drawback, i.e., their low water-resistance properties, because starch is a hydrophilic substance. Many researchers have attempted to solve this problem, mainly by combining additives with the starch matrix, such as lipids^11^, cellulose nanocrystals^12^, and nano-clays^13^. In this study, sunflower seed oil (SSO) and guar gum (GG) were used as supplements to enhance the water-resistance properties of MBS-based films. The incorporation of SSO into the MBS suspension can confer excellent water-resistance properties to films because this oil has a hydrophobic nature. However, the mixture of the MBS suspension and SSO is heterogeneous and immiscible. Accordingly, the incorporation of SSO into the MBS suspension results in an oil-in-water emulsion state, with aggregation of SSO particles and the separation of the SSO/MBS emulsion into MBS and SSO layers. Therefore, GG was supplemented to the filmogenic emulsion as a thickener to impart viscosity and stability. The thickened filmogenic emulsion obtained from the addition of GG trapped the SSO droplets well within its matrix in a dispersed state.

Consequently, the objectives of this study were to manufacture MBS-based edible films using GG and SSO as supplements to enhance their water-resistance properties. We also aimed to determine the mechanical, optical, structural, morphological, and oxygen-barrier properties of the films, including water-resistance properties. The MBS-based edible films reported here can be used as a reference material to develop films or emulsions that can be used in real food systems for wrapping, coating, and attaching to the foods.

## Materials and methods

### Materials

MBS was purchased from the Sung Jin Food Co., Ltd. (Gwangju, Korea). GG (extra-pure grade) was obtained from the Duksan Pure Chemical Co., Ltd. (Ansan, Korea). SSO was purchased from the Daesang Co. (Seoul, Korea). Glycerol (food grade) was provided by LG Household & Healthcare (Seoul, Korea). Calcium chloride dihydrate (chemically pure grade) was supplied by the Daejung Chemicals & Metals Co., Ltd. (Siheung, Korea).

### Formation of SSO/GG/MBS composite films

**Figure 1:**
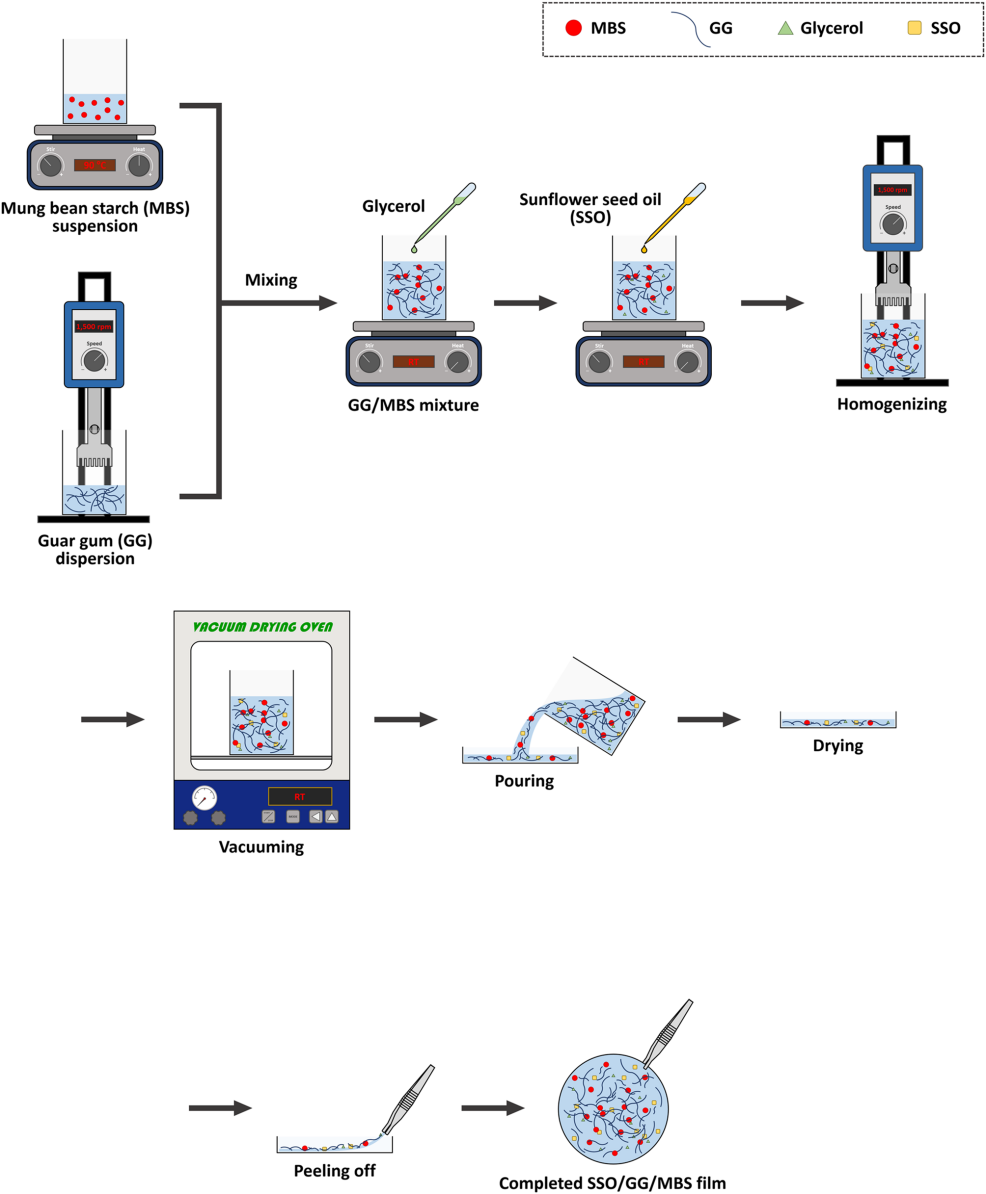
Scheme of the procedure to prepare SSO/GG/MBS composite films. *GG* guar gum, *MBS* mung bean starch, *SSO* sunflower seed oil.

SSO/GG/MBS composite films were prepared as shown in Fig. 1. An MBS suspension (4%, w/w) was prepared with distilled water and heated at 90–95 ℃ by stirring for 30 min. The MBS suspension was then cooled to room temperature (RT; 22 ± 3 °C) with mild stirring. A GG dispersion (1.5%, w/w) in distilled water was manufactured using a high-speed homogenizer (SR30; M-TOPS Co., Ltd., Yangju, Korea) at 15,000 rpm at RT until agglomeration of the GG was completely broken. Subsequently, the prepared MBS suspension and GG dispersion were blended and stirred at RT for 45 min. Glycerol [30% (w/w) of the solid content of the mixture of MBS suspension and GG dispersion (GG/MBS mixture)] was then added to GG/MBS mixture and stirred at RT for 15 min. SSO was added to the resulting mixture at various concentrations [0, 0.5, 1, and 2% (w/w) of the weight of GG/MBS mixture] and homogenized using a high-speed homogenizer (SR30; M-TOPS Co., Ltd.) at 15,000 rpm at RT for 3 min. To remove air bubbles, the filmogenic emulsions were degassed in a vacuum chamber (VS-1202V5; Vision Scientific Co., Ltd., Bucheon, Korea) at RT for 30 min. The films were elaborated using the casting technique. The degassed filmogenic emulsions (9.82 mg of solids cm^−2^) were gently poured over square Petri dishes (polystyrene, 12.5 × 12.5 cm) resting on a leveled surface and dried at 40 °C and 25% relative humidity (RH) for 24 h. The same dry matter density of filmogenic emulsions was dispensed to obtain a constant film thickness. The dried films were carefully peeled off the casting dishes and conditioned at 25 °C and 50% RH for at least 48 h in a thermo-hygrostat (Labmade 011; Sejong Scientific Co., Ltd., Bucheon, Korea) before the analysis of their properties.

### Characterization of the composite films

#### Thickness and mechanical properties

The thickness (μm) of the films was measured using a digimatic indicator (ID-C112X; Mitutoyo Corp., Kanagawa, Japan) with a precision of 0.001 mm at 10 random positions on the film.

The specimens used for measuring the mechanical properties of the films were cut into 25.4 × 100 mm rectangles. The tensile strength (TS) and elongation at break (EB) parameters of each film were investigated using a universal testing machine (Instron 3366; Instron Engineering Corp., Norwood, MA, USA) operated with a load cell of 5 kN, an initial grip separation of 50 mm, and a cross-head speed of 500 mm min^−1^, according to the ASTM D882-02 standard test method. For each film sample, five specimens were measured.

#### Optical properties

##### Color

The color of the films was assessed using a colorimeter (CR-400; Konica Minolta Sensing, Inc., Osaka, Japan) that was calibrated using a standard white plate (*Y* = 93.8, *x* = 0.3131, and *y* = 0.3191). The films were placed on the standard white plate, and the CIE L^*^a^*^b^*^ values were recorded at five random locations on the film surface. The total color difference ($\Delta$E^*^), hue angle (*H*°), and chroma (C^*^) were calculated using the following equations (Eqs. 1–3).

1 $${\Delta E}^{*}=\sqrt{{\left({\Delta L}^{*}\right)}^{2}+{\left({\Delta a}^{*}\right)}^{2}+{\left({\Delta b}^{*}\right)}^{2}}$$

where $${\Delta L}^{*}={{L}^{*}}_{sample}-{{L}^{*}}_{control}$$, $${\Delta a}^{*}={{a}^{*}}_{sample}-{{a}^{*}}_{control}$$, and $${\Delta b}^{*}={{b}^{*}}_{sample}-{{b}^{*}}_{control}$$.

2 $$\begin{gathered} H^\circ = {\tan}^{ - 1} \left( {b^{*} /a^{*} } \right),\quad {\text{when }}a^{*} > 0{\text{ and }}b^{*} > 0 \hfill \\ H^\circ = 180^\circ + {\tan}^{ - 1} \left( {b^{*} /a^{*} } \right),\quad {\text{when }}a^{*} \, < \,0 \hfill \\ H{{^\circ }} = 360{{^\circ }} + tan^{( - 1)} (b^{*} /a^{*} ),\quad {\text{when }}a^{*} \, > \,0{\text{ and }}b^{*} \, < \,0 \hfill \\ \end{gathered}$$

3 $${C}^{*}=\sqrt{{\left({a}^{*}\right)}^{2}+{\left({b}^{*}\right)}^{2}}$$

##### Transparency

The transparency of the films was determined by measuring their transmittance at 600 nm (*T*_600_) using an ultraviolet (UV)–visible spectrometer (UV Mini-1240; Shimadzu Co., Kyoto, Japan), according to the ASTM D1746-92 standard test method. The films were cut into 25 × 40 mm pieces and inserted into a film holder. Five transmittance values were recorded, and the transparency was calculated according to Eq. (4):

4 $$\mathrm{Transparency}\left(\mathrm{\%\it T/\mathrm m \mathrm m}\right)=(\mathrm{log}{T}_{600})/x$$

where *T*_600_ is the fractional transmittance at 600 nm and *x* is the film thickness (mm).

#### Structural properties

##### Fourier transform infrared (FT-IR) spectroscopy

The FT-IR spectra of pure SSO and GG/MBS and SSO/GG/MBS composite films were obtained using an FT-IR spectrometer (Cary 630; Agilent Technologies, Inc., Santa Clara, CA, USA) in the attenuated total reflectance mode. All spectra were recorded in the range of 4,000–600 cm^−1^ at a resolution rate of 4 cm^−1^ by co-adding 128 scans.

##### X-ray diffraction (XRD)

The XRD patterns of GG and MBS granules and GG/MBS and SSO/GG/MBS composite films were obtained using an X-ray diffractometer (X'Pert PW3040/00; Malvern Panalytical Ltd., Worcestershire, Malvern, UK) equipped with Cu Kα radiation (λ = 1.5406 Å). The XRD patterns were recorded at a step size of 0.0170°, a scan step time of 45.72 s, and a scan rate of 2° min^−1^, operated at a voltage of 40 kV and a current of 30 mA.

#### Morphological properties

The film surface and cross-section morphologies were characterized using a scanning electron microscope (SEM) (SU-70; Hitachi Ltd., Tokyo, Japan). The films samples were cut by a razor blade and coated with platinum for 70 s and observed with an accelerating voltage of 20 kV.

#### Oxygen transmission rate (OTR)

The OTR of the films was examined using an oxygen permeation analyzer (8003; Systech Instruments Ltd., Illinois, Johnsburg, IL, USA), according to the ASTM D3895-05 standard test method at 23 °C and 0% RH. The specimens were mounted onto the diffusion cells and exposed to pure oxygen flow on one side, with pure nitrogen flow on the other side. The area of exposure during the test was 56.745 cm^2^ for each sample. Measurements were performed in duplicate and are expressed as the quantity of oxygen molecules passing through the 1 m^2^ of the film surface area over 1 day.

#### Water-resistance properties

##### Preparation of commercial starch-based edible film

To compare water-resistance properties of SSO/GG/MBS composite films with commercial edible film, starch-based edible film on the market (Oblate; Daewha Food, Gimpo, Korea) was also prepared. The oblate film was made of potato starch, corn starch, and soybean phospholipid, and its thickness was 36.10 ± 1.37 µm.

##### Moisture content

Films were cut into square specimens of 20 × 20 mm, and initial weight was measured. Subsequently, the films were dried in a drying oven (VS 41720; Vision Scientific Co., Ltd., Daejeon, Korea) at 105 °C for 24 h. The moisture content was calculated using Eq. (5) as follows:

5 $$\mathrm{Moisture\,content }\left(\mathrm{\%}\right)=\left[\left({W}_{w}-{W}_{d}\right)/{W}_{w}\right]\times 100$$

where *W*_w_ was the initial weight of the film and *W*_d_ was the constant weight of the film after drying at 105 °C for 24 h. Five replicates were performed for each film.

##### Water solubility

The films (20 × 20 mm) were dried in the drying oven (VS 41720; Vision Scientific Co., Ltd.), and the weight of the dried films was determined. Subsequently, the dried films were immersed in 50 mL of distilled water at 25 °C for 24 h. The wet films were re-dried in the drying oven (VS 41720; Vision Scientific Co., Ltd.) at 105 °C for 24 h to obtain their final dry mass. The water solubility of the films was calculated using Eq. (6) as follows:

6 $$\mathrm{Water\,solubility }\left(\mathrm{\%}\right)=\left[\left({W}_{i}-{W}_{f}\right)/{W}_{i}\right]\times 100$$

where *W*_i_ and *W*_f_ were the initial and final dry weight of the films, respectively.

##### Water vapor permeability (WVP)

WVP of the packaging films in one of their most significant properties to determine their fitness for use as packaging materials^14^. In addition, WVP is an important property to characterize the overall water-resistance properties of the films^15^. Therefore, the WVP was considered as the representative attribute in the water-resistance properties of the SSO/GG/MBS composite films. To study the effect of storage time on the WVP in the SSO/GG/MBS composite films, films were stored at 25 °C and 50% RH for 14 days in a thermo-hygrostat (Labmade 011; Sejong Scientific Co., Ltd.). The WVP values in the films were analyzed on days 0, 2, 5, 9, and 14.

WVP was determined gravimetrically according to ASTM E96-05 standard test method. Films cut into circles with a diameter of 65 mm were mounted onto poly(methyl methacrylate) cups (diameter, 45 mm) previously filled with 15 g of calcium chloride dehydrate, with the smooth and shiny side of the film facing outward. Three cups for each film type were weighed and stored in the thermo-hygrostat (Labmade 011; Sejong Scientific Co., Ltd.) at 25 °C and 50% RH for 24 h. Subsequently, the final weight of the cups was measured, and the WVP of the films was estimated from Eq. (7) as follows:

7 $$\mathrm{WVP}\ (\mathrm{g}\cdot \mathrm{mm}/{\mathrm{m}}^{-2}\cdot \mathrm{day}\cdot kPa)=(\Delta W\cdot x)/(A\cdot t\cdot\Delta P)$$

where $\Delta$*W* is the variation in the weight of the cup (g) after 24 h, *x* is the thickness of the film (mm), *A* is the exposed permeation area (0.00159 m^2^) of the film, *t* is the elapsed time (1 day), and $\Delta$*P* is the water vapor partial pressure differences across the film.

##### Wettability of the film surface

To analyze the surface wettability of the films, the water contact angle on the surface of the films was measured using the sessile drop method and a drop shape analyzer (DSA25; KRÜSS GmbH, Hamburg, Germany). A droplet of distilled water (3 μL) was formed at the tip of a syringe needle and carefully deposited onto the top of the surface of the films at RT. The measurements were repeated at three different points on each film sample.

### Statistical analysis

Data analyses were carried out using Statistical Package for the Social Sciences (SPSS) software version 25.0 (SPSS, Inc., Chicago, IL, USA). The significant differences (*P* ≤ 0.05) among result values in the SSO/GG/MBS films in the measurements of their characteristics [thickness, mechanical properties (tensile strength and elongation at break), optical properties (L^*^, a^*^, b^*^, ΔE^*^, *H*°, C^*^, and transparency), OTR, water-resistance properties (moisture content, water solubility, WVP, and wettability of the film surface)] were identified using one-way analysis (ANOVA) followed by Duncan’s multiple range tests. All data are presented as mean ± standard deviation from a minimum of three replicates.

## Results and discussion

### Thickness and mechanical properties

The resulting thickness and mechanical properties of the GG/MBS and SSO/GG/MBS composite films are presented in Table 1. There were no significant differences (*P* > 0.05) in thickness among all film formulations, because the quantity of filmogenic emulsion of each formulation that was poured onto the plate was calculated to the constant weight of solid content for films with a thickness of 92 μm.

**Table 1:** Mechanical properties of GG/MBS and SSO/GG/MBS composite films.

Composite	Thickness (μm)	Tensile strength (MPa)	Elongation at break (%)
GG/MBS	92.40 ± 2.46^a^	27.21 ± 1.19^d^	78.52 ± 2.18^d^
0.5% SSO/GG/MBS	92.40 ± 1.90^a^	16.35 ± 0.54^c^	51.44 ± 2.73^c^
1% SSO/GG/MBS	92.30 ± 1.89^a^	14.37 ± 0.53^b^	24.67 ± 2.17^b^
2% SSO/GG/MBS	92.90 ± 2.56^a^	10.81 ± 0.59^a^	12.33 ± 1.49^a^

Data expressed as mean ± standard deviation of five replicates.

Different lowercase letters (a–d) in the same column indicate a significant difference (*P* ≤ 0.05) by Duncan’s multiple range test.

*GG* guar gum, *MBS* mung bean starch, *SSO* sunflower seed oil.

TS and EB are essential characteristics of packaging materials^16^. The addition of SSO to the GG/MBS films caused a significant reduction (*P* ≤ 0.05) in TS and EB. Generally, the incorporation of an appropriate level of oils into a film matrix decreases TS but increases EB. This phenomenon can be explained by the plasticizer effect of the oil on the film matrix. The oil incorporated into the film matrix is responsible for the partial substitution of strong intermolecular polymer–polymer interactions with weak intermolecular polymer–oil interactions in the film matrix, resulting in heterogeneity of the film structure, featuring discontinuities^17^. The oil diminishes the cohesion of the polymer network forces and extends the amorphous regions of the film matrix. The decrease in film crystallinity decreases the mechanical strength^18^. Evidence of the increase in the amorphous phase in the SSO/GG/MBS composite films as the SSO content increased is provided in subsection “XRD”. Therefore, the addition of oil to films, to a certain extent, decreases the rigidity and increases the flexibility of the film. This phenomenon is the plasticizing action of oils. However, in this study, both TS and EB were reduced in SSO/GG/MBS composite films compared with those in GG/MBS films. This result can be explained by the surfactant-free effect in oil-in-water type filmogenic emulsion. Oil-in-water emulsions are unstable without any added surfactant, and was separated into oil and water phases if it left with no agitation^19^. Therefore, surfactants are used to stabilize the dispersion state in the oil-in-water emulsions. Surfactants can improve the stability in oil-in-water emulsions by enhancing the compatibility between hydrophilic and hydrophobic phases. However, in our study, the surfactant was not used to fabricate the SSO/GG/MBS composite films. The developed films were edible films, and these were needed to retain stability when ingested by the human body. Therefore, the SSO/GG/MBS composite films did not contain any surfactant materials. Accordingly, in the SSO/GG/MBS composite filmogenic emulsions, blend of the SSO (hydrophobic phase) and GG/MBS mixture (hydrophilic phase) was not adequately occurred because they did not contain no surfactant. Surfactant-free oil-in-water emulsions typically generate much larger oil droplets^20^. This phenomenon caused the discontinuities in the film matrix, and lead to a decrease in tensile strength and elongation at break values^21^. Relatively large amount of SSO that was added to films, which induced SSO agglomeration and caused a considerable local concentration stress^22^. For this reason, EB in SSO/GG/MBS composite films was decreased by the addition of SSO. The results of SEM analysis provided proof of SSO agglomeration in the film matrix. These issues are discussed in detail in subsection “Morphological properties”. Other similar work reported that the surfactant-free edible chitosan films containing 0, 0.5, 1, 2, and 3% (w/w) of bergamot essential oil indicated the 113, 65, 63, 50, and 22 MPa of TS, respectively, and 22, 7, 5.5, 6, and 1.7% of EB, respectively^21^. This tendency is in accordance with our research results. On the other hand, another related study reported that the surfactant (Tween 80) containing sweet potato starch films incorporated with 0, 0.5, 1, and 1.5% (w/w) of oregano essential oil showed the 7.26, 5.20, 3.78, and 2.61 MPa of TS, respectively, and 27.87, 43.35, 47.78, and 55.36% of EB, respectively^23^. The fact that the TS values in the films decreased as the amount of oil increased is in line with our study. However, since their films included a surfactant, the EB values in the films increased as oil content increased. The SSO/GG/MBS composite films were developed as edible films. Therefore, their stiffness and flexibility were not significant factors in determining the quality of the films. If a surfactant is added in the SSO/GG/MBS composite films, it is expected that the EB of the films will increase as the SSO droplets are well dispersed in the film matrix.

### Optical properties

Edible films are applied directly to food surfaces in wrapping or coating form. Therefore, the color and transparency of edible films are significant factors for the visual appearance and consumer acceptance of foods. The color values of our film, including the CIE L^*^a^*^b^*^, *H*°, and C^*^ values, are shown in Table 2. The addition of SSO to the SSO/GG/MBS composite films caused a decrease in the a^*^ values and an increase in the b^*^ values, suggesting that the SSO/GG/MBS composite films have more greenish–yellow tones compared with the GG/MBS film, which was attributable to the slight amber color of SSO. However, to the naked eye, there were no noticeable differences in visual appearance among the film samples.

**Table 2:** Color values of GG/MBS and SSO/GG/MBS composite films.

Composite	L*	a*	b*	ΔE*	*H*°	C*	Transparency (%*T*/mm)
GG/MBS	97.06 ± 0.28^ab^	5.10 ± 0.02^c^	− 2.49 ± 0.03^a^	–	359.55 ± 0.01^a^	16.09 ± 0.00^d^	19.77 ± 0.04^d^
0.5% SSO/GG/MBS	96.92 ± 0.11^a^	5.07 ± 0.04^c^	− 2.31 ± 0.03^b^	0.03 ± 0.01^a^	359.57 ± 0.01^b^	15.52 ± 0.21^c^	17.86 ± 0.03^c^
1% SSO/GG/MBS	97.35 ± 0.09^c^	4.94 ± 0.05^b^	− 2.23 ± 0.04^c^	0.10 ± 0.03^b^	359.58 ± 0.01^b^	14.67 ± 0.24^b^	16.66 ± 0.06^b^
2% SSO/GG/MBS	97.28 ± 0.15^bc^	4.77 ± 0.04^a^	− 2.12 ± 0.06^d^	0.16 ± 0.04^c^	359.58 ± 0.01^b^	13.65 ± 0.12^a^	14.70 ± 0.21^a^

Data expressed as mean ± standard deviation of five replicates.

Different lowercase letters (a–d) in the same column indicate a significant difference (*P* ≤ 0.05) by Duncan’s multiple range test.

*GG* guar gum, *MBS* mung bean starch, *SSO* sunflower seed oil.

**Figure 2:**
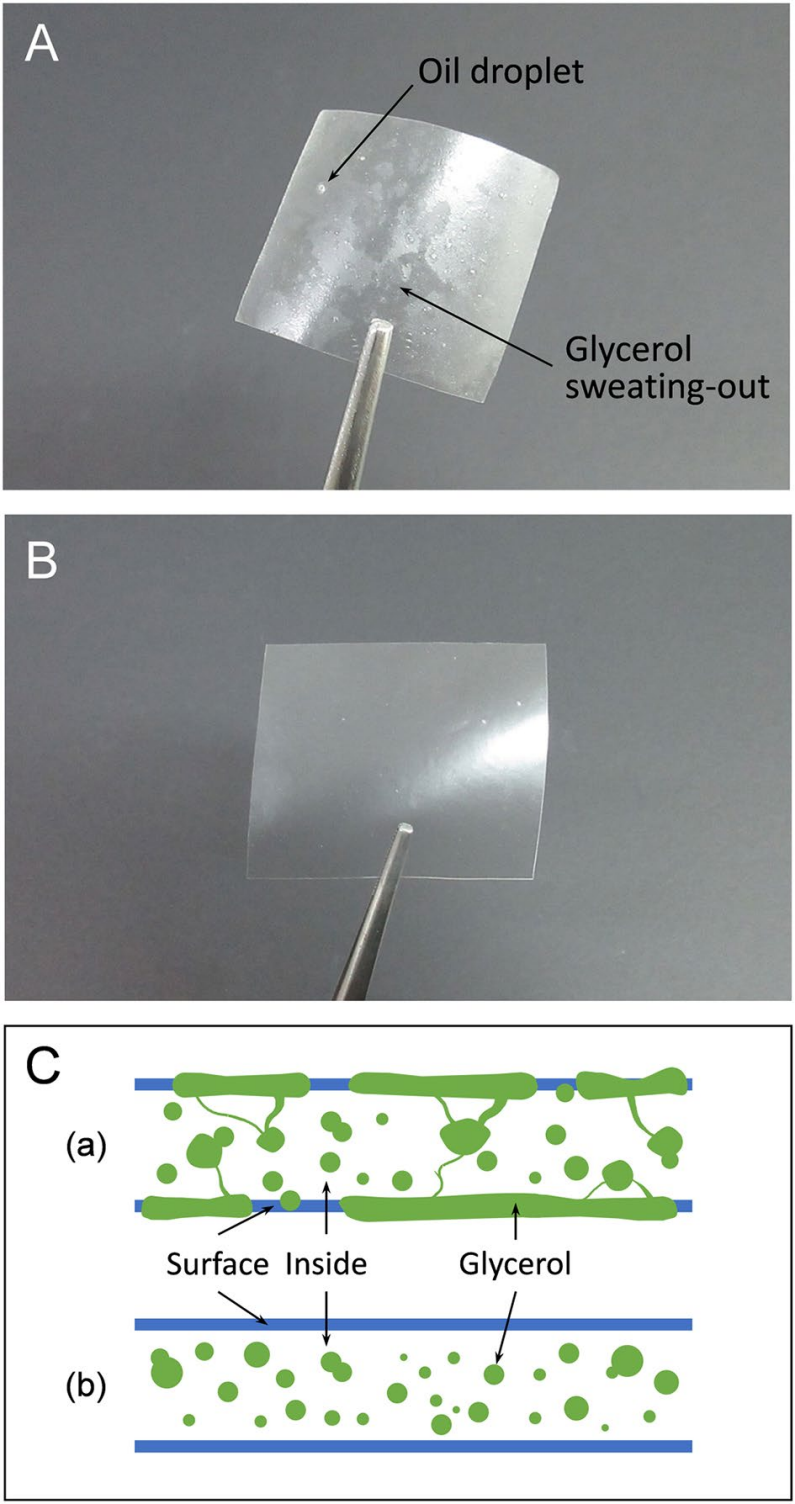
Digital photographs of surface appearance of (**A**) 2% SSO/GG/MBS and (**B**) GG/MBS films; (**C**) schematic cross-section of (**a**) 2% SSO/GG/MBS and (**b**) GG/MBS films. *GG* guar gum, *MBS* mung bean starch, *SSO* sunflower seed oil.

The transparency of the films decreased significantly as the amount of SSO increased. This phenomenon resulted from the increase in diffuse reflectance caused by light scattering in the oil droplet within the film structure^11^. In addition, sweating-out (or exudation) of glycerol from the film matrix to the surface may also affect the decrease in transparency of the SSO/GG/MBS composite films. Figure 2A,B show the surface appearance of 2% SSO/GG/MBS (sample with the maximum amount of SSO added) and GG/MBS (sample without SSO) films, respectively. As shown in Fig. 2A,B, sweating-out of the glycerol from the inside to the surface of the 2% SSO/GG/MBS film was demonstrated with the naked eyes. The intermolecular MBS-GG interactions were diminished as SSO content increased in the SSO/GG/MBS composite films. Therefore, the glycerol dispersed inside the film matrix in the 2% SSO/GG/MBS film was not immobilized and migrated to the surface of the film. On the contrary, there are no sweating-out of the glycerol in the GG/MBS film, since the intermolecular MBS–GG interactions were adequately formed in the film matrix. As a result, the glycerol in the GG/MBS film was well dispersed and entrapped inside the matrix. The behavior of glycerol in the 2% SSO/GG/MBS and GG/MBS films was presented schematically in Fig. 2C,a,b, respectively. The sweating-out phenomenon of glycerol will be mentioned in later subsections “OTR” and “Wettability of the film surface”. Meanwhile, Gao et al.^24^ and Phupoksakul et al.^25^ also reported the decrease in transparency of the film by sweating-out of glycerol. The reduction in transparency (or increase in opacity) of SSO/GG/MBS composite films could be advantageous, as opaque films can effectively block UV radiation. UV exclusion is important for prolonging the shelf-life of lipid-rich foods, which are vulnerable to the oxidative degradation catalyzed by UV radiation^26^.

### Structural properties

#### FT-IR spectroscopy

**Figure 3:**
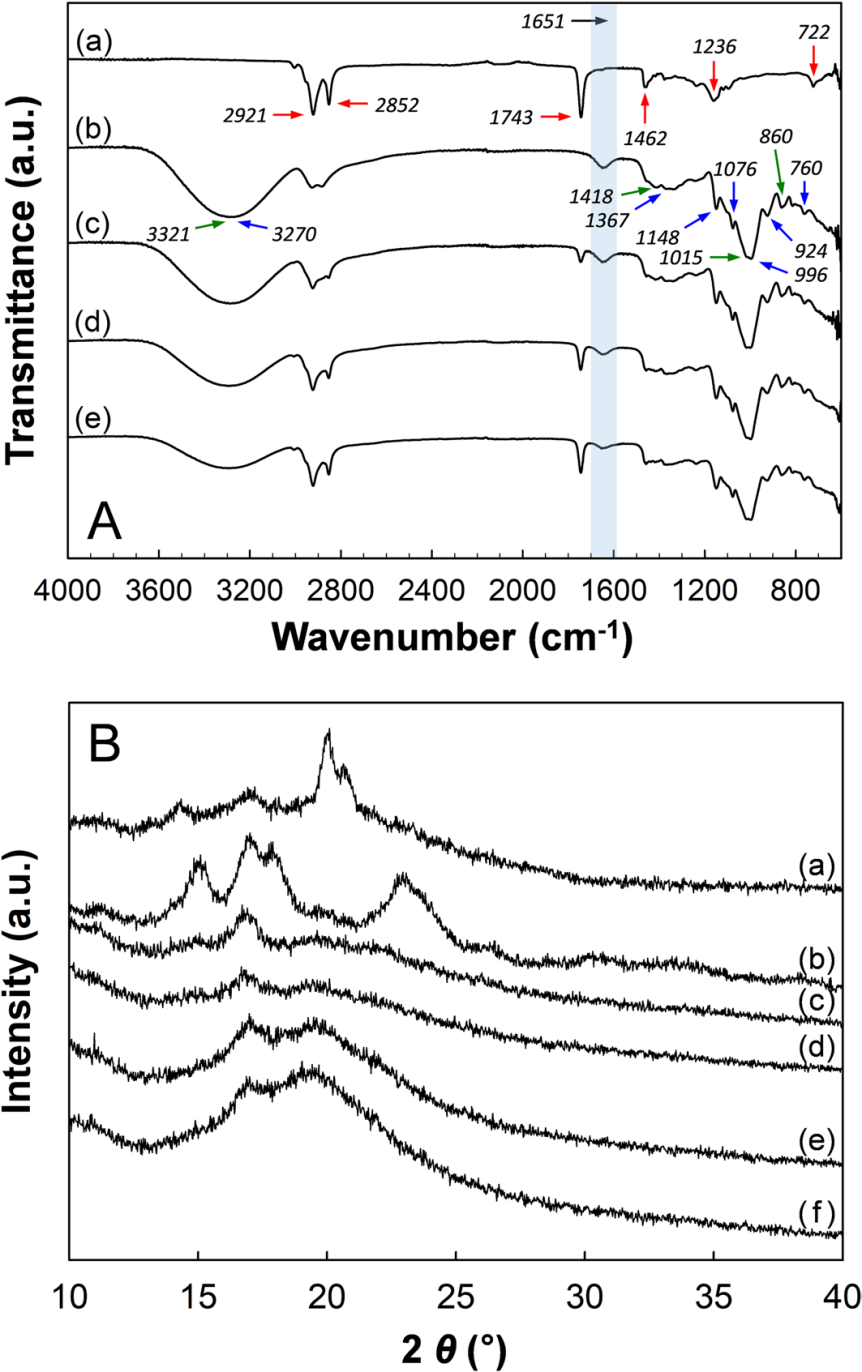
(**A**) FT-IR spectra of (**a**) SSO, (b) GG/MBS film, (**c**) 0.5%SSO/GG/MBS film, (**d**) 1%SSO/GG/MBS film, and (**e**) 2% SSO/GG/MBS film. Peaks marked with red, blue, and green arrows derived SSO, GG, and MBS, respectively; (**B**) XRD patterns of (**a**) GG granules, (**b**) MBS granules, (**c**) GG/MBS film, (**d**) 0.5% SSO/GG/MBS film, (**e**) 1% SSO/GG/MBS film, and (**f**) 2% SSO/GG/MBS film. *GG* guar gum, *MBS* mung bean starch, *SSO* sunflower seed oil.

The FT-IR spectra of SSO, GG/MBS films, and SSO/GG/MBS composite films are plotted in Fig. 3A. The spectra of SSO (Fig. 3A,a) exhibited a broad band at 3,004 cm^−1^ that was attributed to =CH stretching. The two peaks detected at 2,921 and 2,852 cm^−1^ are assigned because of the CH stretching. The peaks detected at 1,743 and 1,462 cm^−1^ represented the ester C=O stretching of the triglyceride and CH deformation, respectively. The peak observed at 1,236 cm^−1^ indicated the bending of CH_2_ groups. The peak 722 cm^−1^ was related to a C–H out-of-plane deformation^27^. These peaks, which were derived from SSO, were detected in all SSO/GG/MBS composite films, and their intensity increased with SSO concentration. The spectra of the GG/MBS film (Fig. 3A,b) indicated the intrinsic peaks of starch and gum. The peaks detected at 3,270 (O–H stretching), 1,367 (C–H bending), 1,148 (C–O stretching), 1,076 (C–O stretching), 996 (C–O stretching), 924 (C–O stretching), and 760 (C–C stretching) cm^−1^ were derived from the MBS^7,28,29^. The peaks observed at 3,321 (O–H stretching), 1,418 (C–H stretching), 1,015 (CH_2_–O–CH_2_ bending), and 860 (galactose and mannose) cm^−1^ originated from the GG^30,31^. The bands at 1,651 cm^−1^ were attributed to the moisture absorbed in the amorphous regions of the films. Therefore, the intensity of this peak indicates the moisture content, which exhibited a decreasing trend with the increasing content of SSO^7^. This interpretation was consistent with the results of the moisture content analysis of the films (subsection “Moisture content”). Compared with the spectra of the GG/MBS film (Fig. 3A,b) and of SSO/GG/MBS composite films (Fig. 3A,c–e), there was no peak shifting, appearance of a new peak, or disappearance of a peak related to GG/MBS film matrix, with the exception of the peaks from the SSO. Therefore, it can be assumed that the incorporation of SSO did not affect the structure of the GG/MBS film.

#### XRD

An XRD analysis was performed to investigate the effect of SSO on the crystalline structure of the SSO/GG/MBS composite films and the compatibility among the components of the films. Figure 3B presents the XRD patterns of the original materials, MBS and GG, in granule form; of the GG/MBS film; and of the SSO/GG/MBS composite films. The pattern of GG granules (Fig. 3B,a) showed three distinct peaks at 2θ = 14.3°, 17.1°, and 20.0°. In contrast, MBS granules (Fig. 3B,b) exhibited the characteristic peaks at 2θ = 15.1°, 17.0°, 18.0°, 22.9°, and 26.4°. This indicated that MBS has a C-type diffraction pattern; i.e., it contains both A- and B-type crystals^32^. The diffraction patterns of the SSO/GG/MBS films were similar to that of the GG/MBS film without SSO. The diffractograms of all SSO/GG/MBS composite films revealed a partially crystalline nature and indicated somewhat weak diffraction peaks at approximately 2θ = 16° and 20°, which were derived from MBS and GG, respectively. This result suggests the existence of intermolecular interactions between the two polymers, i.e., a good compatibility between MBS and GG. The weak peaks observed in the diffraction pattern of the GG/MBS film (Fig. 3B,c) compared with those of the MBS and GG granules (Fig. 3B,a,b, respectively) indicated that the crystal structures of MBS and GG were destroyed during the film preparation. It suggested the good compatibility of the MBS and GG in the film matrix. In addition, the decrease in the crystallinity of the films was also caused by the plasticizer effect of glycerol. The glycerol incorporated in the matrix of the films was formed the intermolecular hydrogen bondings between MBS and GG. Therefore, the crystallinity of the GG/MBS film was decreased compared to the MBS and GG granules, and caused the increase in the chain mobility of MBS and GG^33,34^. Conversely, the peak intensity in the SSO/GG/MBS composite films (Fig. 3B,d–f) was decreased as SSO content increased, which suggests that the addition of SSO in the film matrix led to the formation of a less crystalline structure, thus causing a decrease in the mechanical strength of SSO/GG/MBS composite films. Similarly, Valenzuela et al.^35^ also reported that the incorporation of SSO into a quinoa protein/chitosan film matrix induced a decrease in crystallinity. The reduction in the crystallinity of the SSO/GG/MBS composite films could be attributed to intermolecular interactions between the two polymers and SSO. Therefore, the SSO also increased the chain mobility of the MBS and GG, like glycerol. In other words, not only glycerol but also SSO was acting as a plasticizer in the film matrix. Meanwhile, the increase in the chain mobility in the GG/MBS and SSO/GG/MBS composite films was also affected to the OTR of the films. This issue is discussed in later subsection “OTR”.

### Morphological properties

**Figure 4:**
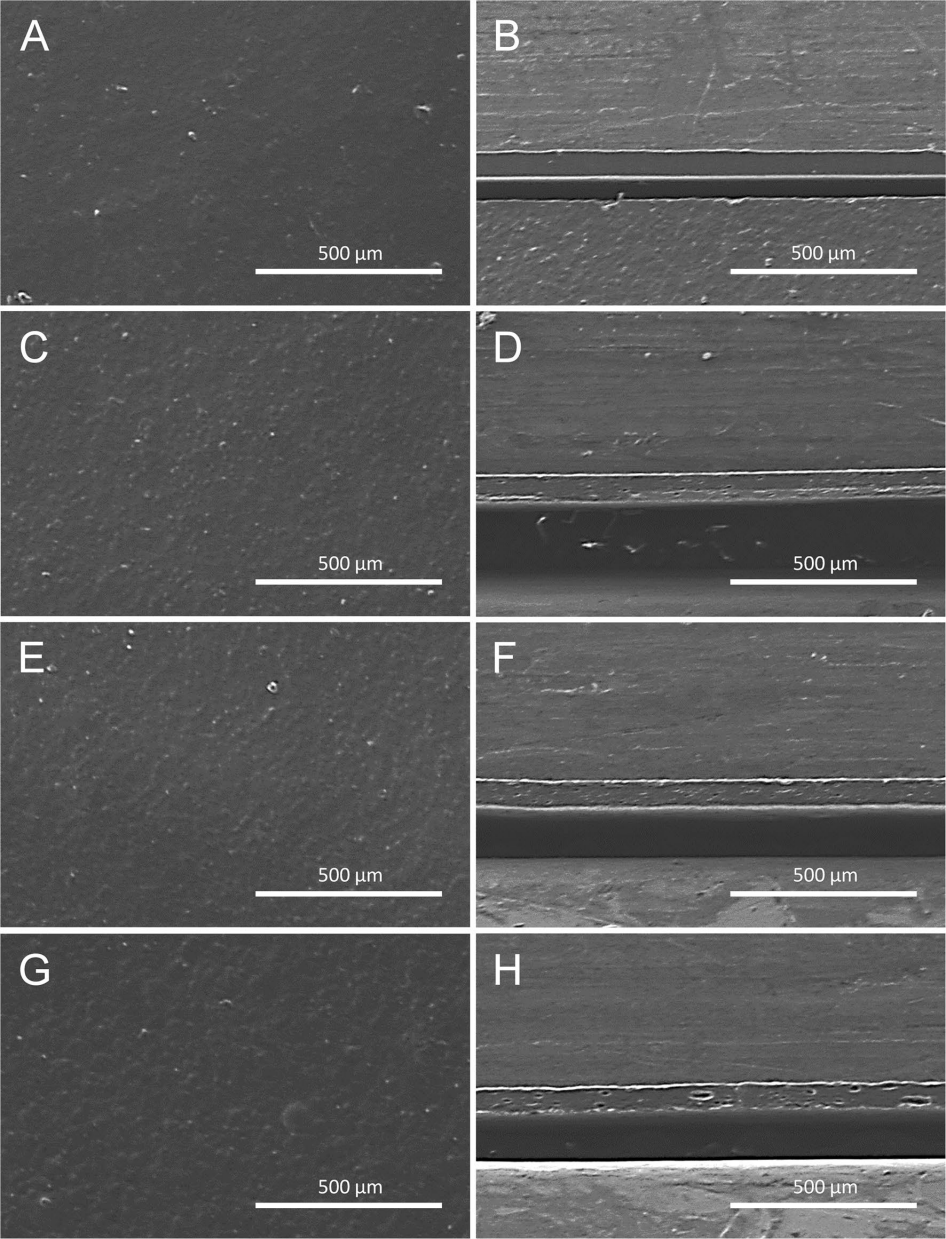
Morphology of GG/MBS and SSO/GG/MBS composite films. (**A**,**C**,**E**,**G**) Surface SEM images of GG/MBS, 0.5% SSO/GG/MBS, 1% SSO/GG/MBS, and 0.5% SSO/GG/MBS, respectively; (**B**,**D**,**F**,**H**) cross-sectional SEM images of GG/MBS, 0.5% SSO/GG/MBS, 1% SSO/GG/MBS, and 0.5% SSO/GG/MBS, respectively. Scale bar is 500 μm. *GG* guar gum, *MBS* mung bean starch, *SSO* sunflower seed oil.

An SEM analysis was carried out to study the formation and distribution of the oil droplet in the film matrix containing SSO. Figure 4 presents the SEM images corresponding to the surface and cross-section of the films. The structure of the GG/MBS film (Fig. 4A,B) without the addition of SSO was compact, homogeneous, continuous, and without pores. Therefore, the GG/MBS film had superior TS and EB values. In contrast, the SSO/GG/MBS composite films exhibited a rough surface and heterogeneous film structure caused by the addition of SSO. The roughness and heterogeneity of the film increased with the increasing concentration of SSO. As shown in SEM images of 0.5% SSO/GG/MBS (Fig. 4C,D) and 1% SSO/GG/MBS (Fig. 4E,F) films, the droplet size was relatively small until the addition of 1% SSO to the GG/MBS films. These results indicate that the SSO was well dispersed in the film matrix. However, the 2% SSO/GG/MBS film (Fig. 4G,H) had larger and fewer lipid globules, which trapped the SSO at both the film surface and interior. This was because of the poor dispersion of SSO droplets in the film matrix at a concentration of 2%, from the low stability of the filmogenic emulsion^36^. Therefore, the aggregation of SSO was accelerated in the film matrix during the drying process. Such an increase in film heterogeneity and decrease in miscibility caused by SSO addition were related to the degradation of the mechanical properties of the films.

### OTR

The OTR values of the films are listed in Table 3. The incorporation of SSO into films caused a significant increase (*P* ≤ 0.05) in OTR because oxygen has a hydrophobic nature and combines well with oil. The SSO in the matrix of the SSO/GG/MBS composite films improved the oxygen dissolution, which accelerates the transfer of oxygen molecules^37^. In addition, the plasticizing effect of SSO also increased the OTR values in the SSO/GG/MBS composite films. The plasticizers reduce the resistance properties of the films to gas transmission by increasing the molecular mobility of the GG and MBS^38^. Therefore, SSO/GG/MBS composite films had significantly higher (*P* ≤ 0.05) OTR values compared with the GG/MBS film without SSO addition. However, there was no significant difference (*P* > 0.05) in OTR values between 1 and 2% SSO/GG/MBS films, despite the difference in the amount of SSO added. This result might be attributed to the effect of sweating-out of glycerol in the SSO/GG/MBS composite film (Fig. 2A). The sweating-out phenomenon of glycerol was also discussed in the previous subsection “Optical properties”. As glycerol is a hydrophilic substance, it was present on the surface of 2% SSO/GG/MBS films, which disturbed the oxygen dissolution and penetration into the film. Accordingly, despite its higher SSO content, the 2% SSO/GG/MBS film did not exhibit a significantly higher (*P* > 0.05) OTR value compared with the 1% SSO/GG/MBS film.

**Table 3:** Oxygen transmission rate (OTR) of GG/MBS and SSO/GG/MBS composite films.

Composite	OTR (cc/m^2^·day)
GG/MBS	0.16 ± 0.00^a^
0.5% SSO/GG/MBS	7.82 ± 0.06^b^
1% SSO/GG/MBS	8.03 ± 0.06^bc^
2% SSO/GG/MBS	8.28 ± 0.23^c^

Data expressed as mean ± standard deviation of five replicates.

Different lowercase letters (a–c) in the same column indicate a significant difference (*P* ≤ 0.05) by Duncan’s multiple range test.

*GG* guar gum, *MBS* mung bean starch, *SSO* sunflower seed oil.

### Water-resistance properties

#### Moisture content

**Figure 5:**
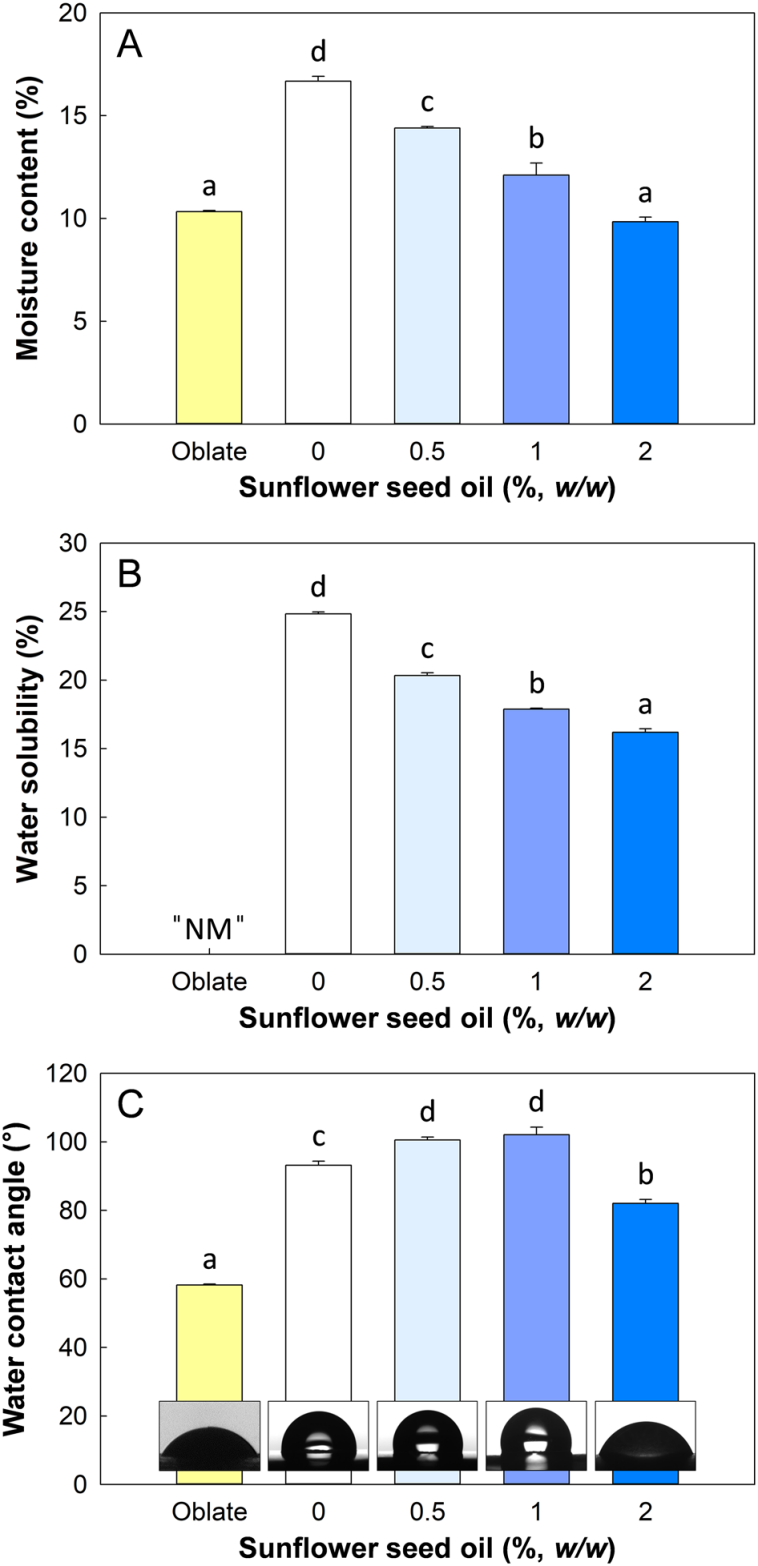
(**A**) Moisture content, (**B**) water solubility, and (**C**) water contact angle of oblate, GG/MBS, and SSO/GG/MBS composite films. Data are expressed as mean ± standard deviation of three replicates. Different lowercases (**a**–**d**) indicate significant differences (*P* ≤ 0.05) by Duncan’s multiple range tests. *NM* not measurable because of disintegration of the film in water. *GG* guar gum, *MBS* mung bean starch, *SSO* sunflower seed oil.

The moisture content of SSO/GG/MBS composite films decreased significantly (*P* ≤ 0.05) with the increase in SSO content (Fig. 5A). This implies that the addition of SSO to the film matrix reduced the hygroscopicity of the films, because the hydrophobic SSO reduced the water-retention capacity of the films^39^. More specifically, fewer sites were available to bind water in the film matrix because of the increased number of hydrophobic chains^35^. In addition, the relatively decreased level of hydrophilic substances, such as MBS, GG, and glycerol, in the film matrix because of the addition of SSO may also have contributed to this tendency. Meanwhile, the moisture content of the oblate film was not significantly different (*P* ≤ 0.05) with that of the 2% SSO/GG/MBS films. This result was caused by the absence of glycerol in the oblate film. Glycerol is a hygroscopic plasticizer. Therefore, glycerol gives hygroscopic properties to films^40^. However, the oblate film did not include glycerol and any other hygroscopic substances, and its moisture content was relatively low.

#### Water solubility

The water solubility of edible films suggests their integrity in an aqueous condition^41^. The incorporation of SSO into the GG/MBS film led to a significant decrease (*P* ≤ 0.05) in water solubility (Fig. 5B). The addition of SSO to the film matrix at a concentration of 0.5, 1, and 2% (w/w) led to a reduction of 18.12, 27.99, and 34.80% in water solubility, respectively. These results can be attributed to the intermolecular interactions between the components of SSO and the hydroxyl groups of MBS and GG, which would reduce the possibility of interaction between hydroxyl groups and water molecules^42^. Consequently, the addition of SSO led to the enhancement of the water-resistance property of SSO/GG/MBS composite films. However, it was impossible to measure the water solubility of the oblate film because this was disintegrated in the DW after 24 h. This fact that the oblate film was very susceptible to direct contact with moisture against its low moisture content means the oblate film had relatively high water vapor barrier properties, but it had relatively low liquid water barrier properties.

#### WVP

**Figure 6:**
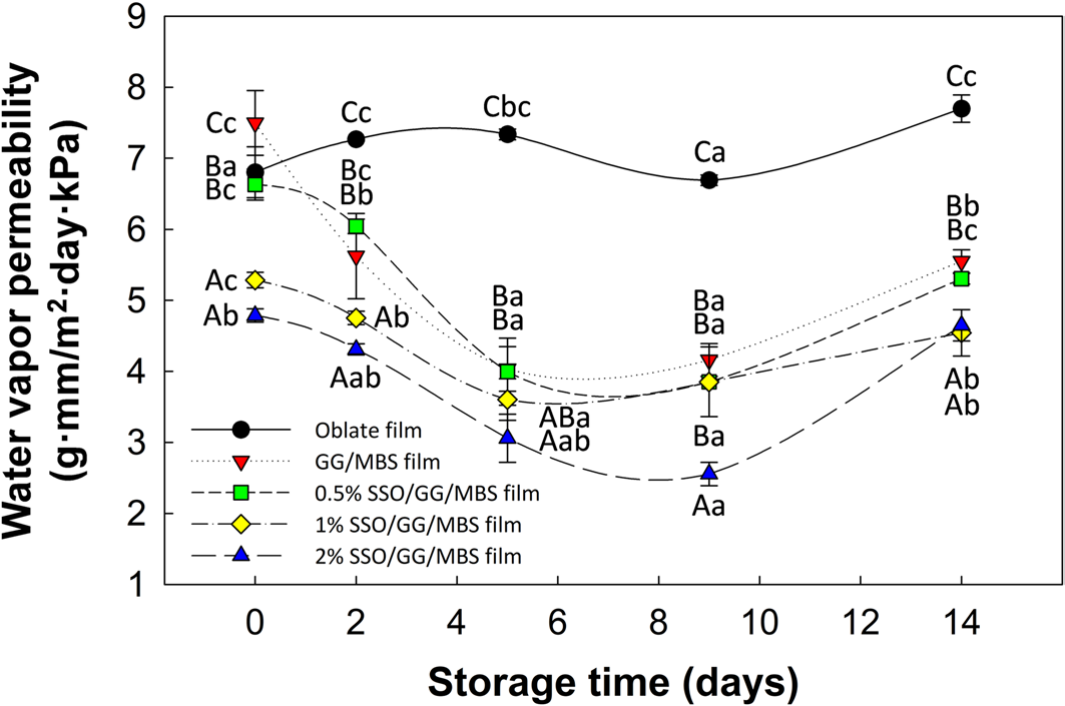
Effect of storage time on water vapor permeability of oblate, GG/MBS, and SSO/GG/MBS films during storage at 25 °C and 50% RH for 14 days. Data are expressed as mean ± standard deviation of three replicates. Different uppercase letters (**A**–**C**) indicate significant differences (*P* ≤ 0.05) among the different groups at the same time points, and different lowercase letters (**a**–**c**) represent significant differences (*P* ≤ 0.05) among time points in the same group by Duncan’s multiple range tests. *GG* guar gum, *MBS* mung bean starch, *SSO* sunflower seed oil.

The various edible films that are prepared using polysaccharides, proteins, and lipids have relatively low water-resistance properties^43^. This attribute is a drawback in the food industry as it accelerates the collapse of edible films when foods come into contact with moisture. Therefore, many researchers have focused on the improvement of the water-resistance properties of edible films^8,23,44^. The WVP is an important criterion among the various water-resistance properties of the films, as it indicates the capability of films to manage the highly humid environment at the surface of food and its surroundings. The WVP of the films depends on many various factors such as hydrophilicity or hydrophobicity of polymer materials, mobility of polymer chains, presence of cavities or cracks, structural integrity, and crystalline/amorphous ratio, type and level of plasticizer, thickness, ambient conditions (temperature and RH), and so on^45^. Changes of WVP values in the oblate, GG/MBS, and SSO/GG/MBS composite films during storage time are shown in Fig. 6. The initial WVP values of oblate, GG/MBS, 0.5% SSO/GG/MBS, 1% SSO/GG/MBS, and 2% SSO/GG/MBS films were 6.80, 7.50, 6.63, 5.29, and 4.79 g mm m^−2^ day^−1^ kPa^−1^, respectively. The differences among the samples were attributed to the variation of the addition level of SSO in the films. Lipophilic substances are effective in reducing of WVP in the films^46^. Therefore, the increased level of SSO in the GG/MBS films decreased the WVP of the films. These results are in accordance with those of other studies of the incorporation of oil into hydrophilic biopolymer films^47,48^. The addition of SSO to the GG/MBS film matrix might lead to hydrogen bonding interactions between SSO and the functional groups of GG and MBS. As a result, the hydrophilic portion of the MBS and GG molecules that is available for interaction with water molecules may have decreased^49^. The hydrophobic nature of SSO also led to the decrease in the WVP of SSO/GG/MBS composite films because water vapor permeation generally occurs through the hydrophilic portion of the films^50^. The oblate film showed the significantly high (*P* ≤ 0.05) WVP values than those in the GG/MBS and SSO/GG/MBS composite films all the entire storage period, except for the initial day.

Meanwhile, the GG/MBS and SSO/GG/MBS composite films showed a tendency of the WVP to decrease. However, after 5 or 9 days of storage, the decrease tendency was disappeared, and WVP values in the films began to increase. This could be result of the presence of optimal (or balanced) crystalline/amorphous ratio in each film. It is reported that the barrier properties of the film were maximized when the film matrix forms the optimal crystalline/amorphous ratio. However, the crystalline/amorphous ratio in the starch-based film is constantly increased during the storage period, because of the amorphous-to-crystalline phase transition. Therefore, the WVP was decreased just prior to the crystalline/amorphous ratio in the film matrix was reached at the optimal state, and the lowest WVP values were achieved at the optimal crystalline/amorphous ratio. Since then, the crystalline/amorphous ratio gets out of the optimal state, and the WVP values in the films gradually increased. In other words, the time point when the GG/MBS and SSO/GG/MBS films showed the lowest WVP values was not immediately after fabrication but after 5–9 days of aging (or conditioning). Other researchers, Hernández-Muñoz et al.^51^ and Mali et al.^52^ also reported the similar tendency about the change of WVP in starch-based films during the storage period. On the other hand, the change of the WVP values in the oblate film during the storage time did not indicate the tendency to increase after decrease. This was considered because the composition of the oblate film was simpler than that of the GG/MBS and SSO/GG/MBS composite films.

WVP values in the GG/MBS and 0.5% SSO/GG/MBS films had no significant differences (*P* > 0.05) throughout the storage time. In addition, there were no significant differences (*P* > 0.05) between 1 and 2% SSO/GG/MBS films, except for day 9. These results suggested that the addition of 1% (w/w) SSO in the GG/MBS film was most ideal for the water-resistance properties of the film.

#### Wettability of the film surface

The measurement of the water contact angle is a method that is used to determine the hydrophobicity or hydrophilicity of material surfaces. A high water contact angle implies greater hydrophobicity of the surface, and vice versa. Figure 5C shows the water contact angle of the oblate, GG/MBS, and SSO/GG/MBS composite films. The oblate film had the lowest water contact value among the all film samples. Meanwhile, in the GG/MBS and SSO/GG/MBS composite films, the water contact angle of the film surfaces increased significantly (*P* ≤ 0.05) until the addition of 1% SSO between GG/MBS film and SSO/GG/MBS composite films but was significantly decreased (*P* ≤ 0.05) to the lowest value in the 2% SSO/GG/MBS film. This decrease might have been caused by the migration of the glycerol from the film inside to its surface (sweating-out) in the 2% SSO/GG/MBS films^53^ (Fig. 2A). The SSO molecules decreased the intermolecular MBS–GG interactions considerably in the 2% SSO/GG/MBS film matrix. Therefore, a portion of the glycerol exuded into the film surface, which transitioned into a super-hydrophilic state because glycerol is a hydrophilic substance. For this reason, the water contact angle value was lowest in 2% SSO/GG/MBS films compared with that in all other film samples.

## Conclusions

This study demonstrated that the addition of SSO to the MBS-based film matrix effectively improved the overall water-resistance properties of the film. Notably, the addition of 1% (w/w) of SSO to MBS-based films reduced the water solubility and the WVP of the film at initial day (day 0) by both about 30%, and increased the water contact angle by about 10% compared with the GG/MBS film (without SSO addition). In addition, the 1% SSO/GG/MBS film had no severe side effect of SSO addition, sweating-out of glycerol. Therefore, the 1% SSO/GG/MBS film was chosen as the optimal SSO/GG/MBS composite film as it exhibited excellent overall water-resistance properties. Consequently, the results of our study suggest that the 1% SSO/GG/MBS film reported here has potential as a new edible film for the food-packaging industry. Nevertheless, further studies are required to assess the effect of this film in the real food system.

## Data Availability

The data sets generated during the current study are available from the corresponding author on reasonable request.
